# Identifying Health Status in Grazing Dairy Cows from Milk Mid-Infrared Spectroscopy by Using Machine Learning Methods

**DOI:** 10.3390/ani11082154

**Published:** 2021-07-21

**Authors:** Brenda Contla Hernández, Nicolas Lopez-Villalobos, Matthieu Vignes

**Affiliations:** 1 School of Fundamental Sciences, Massey University, Palmerston North 4442, New Zealand; bhernand@massey.ac.nz; 2 School of Agriculture and Environment, Massey University, Palmerston North 4442, New Zealand; n.lopez-villalobos@massey.ac.nz

**Keywords:** milk spectra, mid-infrared (MIR) spectrometry, cow health, machine learning, neural networks

## Abstract

**Simple Summary:**

Diseases in dairy livestock farming can lead to important economic losses. Several studies have been conducted to identify illness such as lameness by using MIR spectrometry data and relying on partial least squares discriminant analysis. However, this method suffers some limitations. In this study, random forest, support vector machine, neural network, convolutional neural network and ensemble models were used to test the feasibility of identifying cow sickness among 1909 milk sample MIR spectra from Holstein-Friesian, Jersey and Holstein-Friesian × Jersey crossbreed cows. The results obtained show that it is possible to identify a health problem with a reasonable level of accuracy using a neural network.

**Abstract:**

The early detection of health problems in dairy cattle is crucial to reduce economic losses. Mid-infrared (MIR) spectrometry has been used for identifying the composition of cow milk in routine tests. As such, it is a potential tool to detect diseases at an early stage. Partial least squares discriminant analysis (PLS-DA) has been widely applied to identify illness such as lameness by using MIR spectrometry data. However, this method suffers some limitations. In this study, a series of machine learning techniques—random forest, support vector machine, neural network (NN), convolutional neural network and ensemble models—were used to test the feasibility of identifying cow sickness from 1909 milk sample MIR spectra from Holstein-Friesian, Jersey and crossbreed cows under grazing conditions. PLS-DA was also performed to compare the results. The sick cow records had a time window of 21 days before and 7 days after the milk sample was analysed. NN showed a sensitivity of 61.74%, specificity of 97% and positive predicted value (PPV) of nearly 60%. Although the sensitivity of the PLS-DA was slightly higher than NN (65.6%), the specificity and PPV were lower (79.59% and 15.25%, respectively). This indicates that by using NN, it is possible to identify a health problem with a reasonable level of accuracy.

## 1. Introduction

Diseases of dairy cattle cause important economic losses to dairy farmers [1,2,3]. These economic losses are related to the reduced milk production of sick cows, unusable milk, veterinary costs, medicines for treatment and early culling of the cow in some cases. Additionally, several studies have shown that the fertility of the cows is negatively affected by lameness, one of the most common diseases in dairy cattle [4,5]. Hence, the early identification of a disease in a cow, before it can be recognised by farm staff, is vitally important to the farmer.

Analysing the composition of cow milk is a possible cost-effective approach to identify lameness and mastitis in cows. Physiological and behavioural changes in cows associated with lameness are also associated with changes in milk composition [6]. Moreover, changes in milk composition are associated with the metabolic status of cows and their health [7].

Several methods can be applied to determine milk composition, such as gas–liquid chromatography, which is the most accurate method. However, this method is expensive and time-consuming [8]. Mid-infrared (MIR) spectrometry is a low-cost, fast and effective method to predict milk composition [9]. This technique has been widely adopted as a routine test in herds. The data obtained by the MIR can be considered the fingerprint of the chemical bonds presented in the molecules of a milk sample [10]. For this reason, it is a potential tool for identifying diseases by analysing changes in milk composition. It is a non-invasive method and would imply a limited cost or no additional labour for farmers since milk sampling is already performed routinely and spectral data can be available for external analysis.

Many studies have been carried out to identify diseases such as mastitis and lameness by analysing data obtained from MIR spectrometry. Mineur et al. [6] used MIR spectrum data to predict lameness by using partial least squares discriminant analysis (PLS-DA). They obtained 59.8% in sensitivity and 62.5% in specificity. Bonfatti et al. [11] used PLS-DA to predict lameness and obtained 65.7% in sensitivity and 56.1% in specificity for cows which were in early stage of lactation (days in milk ≤ 120) and using the MIR spectrum and animal factors (parity number, age at calving, days in milk, breeding values and type traits). Additionally, Rienesl et al. [7] predicted mastitis from MIR spectra and obtained 60.5% in sensitivity and 70.8% in specificity using PLS-DA in cows, which had the herd-test day and diagnosis of mastitis in a period of ±7 days. These studies focused on using traditional statistical methods, such as PLS-DA, which is used to predict a categorical variable. However, Yuanyuan and Zhibin [12] highlighted that PLS-DA is a linear modelling method, which omits the possible non-linear relationship between the dependent and independent variables in MIR spectra data.

Machine learning methods have been widely used in similar settings [13]. Examples include: detection of pregnancy status [14], bovine tuberculosis status [15], milk quality traits in dairy farms [16] and the risk of developing metritis, hyperketonemia and mastitis after calving by using the prepartum behaviour [17]. These methods can discover complex, latent patterns between predictor variables and the trait of interest—the disease status, in our work—even if this relationship is non-linear. Additionally, these methods do not need to have a preconceived hypothesis compared with other more traditional statistical methods. As a result, they have the potential to improve the accuracy of various investigations. Additionally, each MIR spectrum can be considered a one-dimensional image because it is a fingerprint of the chemical bonds. Machine learning methods are known to accurately handle image processing. The general objective of this study was to test the feasibility of using the data obtained by MIR spectrometry to identify the cow’s health status—a class from a machine learning point of view—by using a series of machine learning techniques including random forest (RF), support vector machine (SVM), neural network (NN), convolutional neural network (CNN) and ensemble models. The efficiency of identifying health status by using these methods was compared to that of PLS-DA.

## 2. Materials and Methods

### 2.1. Data of Animals and Farms

In this study, the data from 467 cows were collected from Dairy 1 and Dairy 4 farms (Massey University, Palmerston North, New Zealand) in the 2016–2017 season. A total of 1909 milk samples were collected during Aug 2016 to May 2017.

The Massey University Dairy 1 farm is managed as a low-intensity production system with 257 cows milked once a day (OAD) throughout the entire season. The feed strategy includes fresh ryegrass (Lolium perenne)–white clover (Trifolium repens) pasture as the main diet component, with restricted supplementation and the sporadic use of grazing crops utilised in summer. The herd consisted of 66 Holstein-Friesian (F), 55 Jersey (J) and 136 F × J crossbred cows. The Massey University Dairy 4 farm, located adjacent to the Dairy 1 farm, is a farm managed as a high-intensity production system with 620 cows milked twice a day (TAD) throughout the season. Ryegrass–white clover pasture is also the main feed source but, in this case, a higher supplementation level is included throughout the year. For this study, spectral data were available only from 51 F and 159 F × J cows. More details of the farms are given in Correa-Luna et al. [18].

The meta-information contained for each milk sample included cow identification, herd-test date, breed of cow, herd, calving date, parity number, days in milk (DIM) (herd-test date–calving date), sampling date of MIR spectrum, percentages of fat, protein and lactose in the milk, somatic cell count, concentration of urea in the milk and the respective MIR spectra data.

Each health record contained cow identification, disease and description of the treatment, if the cow received any antibiotic. The selected health records of cows were those that matched the cow identification provided from the MIR spectra dataset, in a time period of 21 days before and 7 days after milk samples were obtained. Cows in the unhealthy group were diagnosed as early as 21 days before and as late as 7 days after test-day with one or several of the following self-explanatory disease labels: lameness, mastitis, reproductive disorder, calving disorder or other ailments. In the category of reproductive disorders were several subcategories including vaginal discharge, metritis, anoestrous and abnormal cycling. Cows were considered to be in the healthy group if they were not diagnosed with a health problem during the same time window.

### 2.2. MIR Spectra Data

Milk samples from each cow were taken in early (September), mid (December) and late (March) lactation in the two herds. These samples were analysed by MilkTestNZ (Hamilton, NZ) using a CombiFoss™ 7 instrument (Foss Electric, Hillerød, Denmark) and the MIR spectra were extracted for each milk sample. The MIR spectrum for each milk sample contained 1060 absorbance values for wavenumbers equally exposed in the 926 cm^−1^ to 5012 cm^−1^ range region.

### 2.3. Statistical Analysis

All statistical analyses were carried out with the R software version 4.0.3 [19].

#### 2.3.1. Data Pre-Processing

Lasch 2012 [20] mentioned six common methods of data pre-processing, of which four of them are typically used in the practical pre-processing application of MIR spectra. These are (i) quality test, (ii) spectra filtering, (iii) normalisation and (iv) some forms of variable reduction. In this study, however, only spectra filtering and the normalisation method were performed.

The reason that a quality test was not performed was that the datasets had very few sick cases, which could have been removed as outlier records and therefore the analysis could not be done. Ideally, machine learning methods would deal with extreme samples automatically by selecting/weighing the most appropriate variables for classification.

Savitzky–Golay (SG) filtering has been widely used for the spectra filtering method, as it reduces noise from spectrum data without losing important characteristics [21]. According to Lasch 2012 [20], the advantage of using this method is that the derivative calculation and smoothing of the data can be performed in one step. For our study, this consisted of a second derivative with a window size of 15 and an order 2 polynomial, which were the parameters that gave the best overall discriminatory performance. The R package to perform the SG method was prospectr [22].

Additionally, wavenumbers related to water absorbance were removed from the spectra because these bands were found to have high noise levels (from 1600 to 1689 and from 3048 to 3666 cm^−1^). Thus, only 872 potentially meaningful wavenumbers of the spectra were used for subsequent analysis.

For normalisation, the standard variate method was calculated, and the base R package was used.

#### 2.3.2. Calibration Models

Table 1 shows that the distribution of classes (sick vs. non-sick) is unbalanced, as one of the classes contains a smaller number of training observations compared to the other class. Ganganwar 2012 [23] highlighted that in such settings, the accuracy of the models can be good for predicting the majority class. However, the predictions of the minority class can be very poor because the algorithm is mainly influenced by the majority class.

Several solutions were proposed for unbalanced datasets, either at the data or at the algorithmic level [24]. For example, at the data level, oversampling increases the number of observations in the minority class, while undersampling decreases the number of observations in the majority class. Both aim at rendering the dataset balanced. Algorithmic level includes the cost of learning, which takes into account the misclassification cost of one class versus that of the other [23]. This can be achieved by applying the cost in the form of weights into the algorithm [25]. Larger weights are given to the minority class in the loss function. Thus, the algorithm focuses on reducing the errors in the minority class rather than majority class [25].

Due to the very low number of sick cases, the undersampling method was not suitable for the dataset. Japkowicz and Stephen 2002 [26] mentioned that modifying the cost of misclassification of the classes may have the same performance as oversampling without increasing the size of the datasets. Therefore, weights for RF, SVM, NN and CNN were applied in order to balance the data. The initial weights to adjust the models were the inverse of the class proportions, and they were tuned to obtain optimal discrimination.

The data were randomly divided into 80% training and 20% validation samples, ensuring that the validation sample had at least one sick case. In order to evaluate the performance of the models, Monte Carlo cross-validation was used. This process to randomly generate different and independent training and validation samples was repeated ten times, ensuring that there was always at least one sick class case in each sample. Then, for each validation sample, the respective accuracy measures (see Section 2.3.3) were calculated. The average and the standard deviation of each accuracy measure were estimated from the ten repeats.

##### Partial Least Squares Discriminant Analysis (PLS-DA)

We used the mixOmics R package [27] for the implementation of PLS-DA. The optimal number of components was adjusted by considering the area under the curve (AUC) for the identification of the health status.

##### Random Forest (RF)

We used the ranger R package [28] for the random forest analysis. The initial number of trees used for the model was the default number in the ranger function, which is 500. For the initial value of the parameter of *m* (variables to try at each of the splits in the trees), we used the squared root of the total number of variables in the dataset. These parameters were tuned so as to obtain the best discrimination for the identification of the health status. Each forest consisted of 800 trees, with an *m* value of 30, and the weights were 0.0049 and 0.9951 for the for the healthy and sick cases, respectively.

##### Support Vector Machine (SVM)

We performed all the computations of the SVM approach using the e1071 [29] R package. For imaging processing, the best kernel is a polynomial function. More specifically, we used a second-order polynomial as an initial kernel. The kernel and class weights were tuned to optimise the AUC of the identification of the health status. A Gaussian Radial Basis kernel was retained, and the weight for the healthy cases was 0.065 and that for the cases with diseases was 0.935.

##### Neural Network (NN)

We used the keras [30] R package to train the NN, with the help of the reticulate [31] R package, which allows integrated computations in Python and R. The initial parameters for the NN were set according to Chollet and Allaire 2018 [32] (2018). The NN parameters were tuned to obtain an optimal AUC for the identification of the health status. The optimal neural network model consisted in two hidden layers, with 32 and 2 hidden derived features, respectively. The activation function for the first hidden layer was the ReLU function and that of the second hidden layer was a sigmoid function. The loss function used was the Mean Absolute Error, and the weights for the healthy and non-healthy cases were 0.935 and 0.065, respectively.

##### Convolutional Neural Network (CNN)

These models were also implemented with the keras [30] and reticulate [31] R packages. The initial model architecture for the CNN in the dataset was chosen according to the details given in Liu et al. [33]. We ran the CNN using the Adam optimiser during 40 epochs, considering the 20% of the training dataset as validation for internal optimisation. The final parameters were: one convolutional layer with 84 filters and a kernel size of 7 with a Leaky ReLU activation function. Max pooling layer was used, and then the Fully Connected Layer. The loss function that we used was the cross-entropy. The weights were 0.045 and 0.955 for the healthy and the disease cases, respectively.

##### Ensemble Models

Three stacking ensemble models, which are models that combine the outputs of different models to perform the classification [34], were used. They rely on three different meta-classifiers, namely majority vote, averaging and weighted averages. The classifications obtained by RF, SVM and CNN were used in the majority vote. The classification probabilities obtained for CNN and RF were used in averaging. In weighted averaging, the weights of RF and CNN were 0.25 and 0.75, respectively.

#### 2.3.3. Measures of Accuracy

In order to evaluate the performance of the models, a confusion matrix was constructed for each classification problem. The rows represent the predicted cases by the models. The columns represent the real values. The definition of each number in the confusion matrix is the following:

True Positives (TP): The cases that were predicted by the model as sick and were truly sick cases.

False Positives (FP): The cases that were predicted by the model as sick and were non-sick cases.

True Negatives (TN): The cases that were predicted by the model as non-sick and were truly non-sick cases.

False Negatives (FN): The cases that were predicted by the model as non-sick and were sick cases.

By using the confusion matrix, the following metrics were calculated:

Accuracy: This is defined as the proportion of the data that was correctly classified by the model. However, when the data are unbalanced, the results may be over-optimistic [35].

Accuracy = (TP + TN)/(TP + FP + FN + TN)

Sensitivity: It is also known as the True Positive Rate or recall measure and refers to the proportion of sick animals that are correctly identified by the model.

Sensitivity = TP/(TP + FN)

Specificity: This is the ratio of correctly identified negative cases by the model.

Specificity = TN/(FP + TN)

Area under the ROC curve (AUC): The ROC curve is a probability curve and provides an overview of the performance of the models. In classification problems, probabilities are used to be able to classify into categories at a certain threshold. In our study, this was set at 0.5. This means that if the probability was greater than 0.5, the cows were classified as sick cases. On the other hand, if the probability was lower than 0.5, the cows were classified as non-sick cases. The ROC curve plot has on the *x*-axis the False Positive Rate (FPR), which is 1-Specificity. The *y*-axis has the Sensitivity. The AUC is the area that is under the ROC curve, which takes values between 0 and 1. The AUC indicates how well the model can separate positive and negative cases. A higher AUC value represents a better identification between the sick and non-sick cases by the model.

Positive predictive value (PPV): This is the probability that a cow is identified as sick by the model and is sick in reality. It is affected by the prevalence of the disease in the sample.

PPV = TP/(TP + FP)

Negative predictive value (NPV): This is the probability that a cow is identified as non-sick by the model and is actually not sick. Similarly to PPV, this value is influenced by the prevalence of the disease in the sample.

NPV = TP/(TP + FP)

Matthews correlation coefficient (MCC): According to Chicco and Jurman [35], this alternative measure is not influenced by unbalanced data. The author defined it as the calculus of the Pearson product–moment correlation coefficient between the actual and predicted values. The interval of this measure is [−1, +1]. Obtaining MCC near 1 means that the model is giving very accurate predictions. However, an MCC near −1 means that the performance of the model is poor. An MCC equal to zero means no better than a random prediction.

MCC = (TP × TN − FP × NF)/[(TP + FP)(TP + FN)(TN + FP)(TN + FN)]^1/2^

Chicco and Jurman [35] suggested that the MCC value can be projected to an interval of [0, 1]. This is known as normalised MCC (nMCC) and is calculated from the following as nMCC = (MCC + 1)/2, with 0 being the worst scenario and 1 being the best scenario.

## 3. Results and Discussion

The performance of the models for the two-class problem classification of cows that have any health problem and healthy cows is reported in Table 2. The model with the highest sensitivity was the SVM algorithm (66.39%). Nearly two thirds of the cows that were diagnosed with a disease were correctly classified. However, the corresponding PPV was only 13.48%; this is the proportion of truly (as per diagnosis) sick cows that were classified as sick by this model. Thus, SVM had many false positives. Notwithstanding, NN obtained a higher percentage in PPV (59.99%) and the third highest sensitivity value at 61.74%. In terms of specificity (proportion of correctly classified non-sick cows), NN obtained the highest value (97%) among all models, along with a high NPV (97.87%). All models achieved very good NPV values. In addition, NN obtained the highest MCC value, which was 0.57, and AUC (79.37%), indicating good performance.

The summary of the normalised Matthews correlation coefficient (MCC) values, which are the MCC projected to an interval of [0, 1], obtained by the model in the dataset is provided in Table 3. Overall, the NN approach was the best at performing the classification for “any health problem”, followed by the CNN approach.

The main objective of this study was to evaluate the potential to identify any health problem in grazing cows using milk MIR spectra data analysed through different computational methods. Previous studies have sought to identify a specific disease using MIR spectra [6,7,11,15]. However, to the best of our knowledge, our study is the first to evaluate the potential to identify any of lameness, mastitis, reproductive disorder or calving disorder of cows in the same dataset by MIR spectrum analysis.

A model with a high sensitivity value is preferable to one with high specificity in our setting. Our priority was to identify cases of sick cows. However, it is important to achieve a balance between these two measures. If a model has a high sensitivity value but returns many false positives, i.e., cows that are wrongly identified as sick by the model, farmers would be testing many healthy cows. Likewise, if a model has a high specificity value but returns many false negatives, i.e., the model wrongly identifies sick cows as healthy, the farmers would not be able to prevent the disease in time and apply a timely treatment. Animal welfare would be compromised, and potential downstream losses would follow. The MCC measure provided a pragmatic model performance measure without being influenced by the unbalanced distribution of the classes.

Although NN scored the third highest value in sensitivity (61.74% of sick cows were identified correctly), almost 60% of cows classified as sick were truly sick, which is the highest PPV value. Compared to SVM, which had the highest sensitivity value (66.39%) but a poor PPV value of 13.48%, NN is the method of choice, as it has by far fewer false positives. In addition, NN had a higher MCC (0.578) and nMCC (0.789) in identifying any health problem, which shows very good agreement between current and predicted values, indicating that the model performs well.

The very low number of sick cow cases in the dataset was one limitation of this study. The dataset that we used had a prevalence of various diseases (lameness, mastitis, reproductive disorder, calving disorder labels) of 5.40% in the dataset. This made the datasets highly skewed and more challenging to analyse through algorithms. However, this is a dataset that reflects the reality of a grazing dairy cow herd. Therefore, it may be considered a final test for a potential application by farmers with realistic performance measures of the analysis methods to identify illness in dairy cows.

A study was performed separating the data by milking frequency (Dairy 1 with cows milked OAD and Dairy 4 with cows milked TAD). Tables S1 and S2 in the Supplementary Materials show the results obtained from the different models’ performance. The MCC is improved for OAD milked cows but decreases slightly for TAD milked cows. This could be due to the different feeding regimes, environment or even the milking frequency playing the role of a confounder effect. This is known as Simpson’s paradox, which refers to an inversion in the sign obtained by the association of a pair of variables [36]. In other words, when the records of milk samples from sick cows are in the same database without being separated by milking frequency, they seem to indicate that cows are healthy. By separating the data into two different datasets, however (Dairy 1 and Dairy 4), it can be seen that they are not really healthy. The mentioned factors may not be the only confounding variables. The MCC of the dataset that contains cows that milk TAD seems to decrease, although this was a larger database compared to the OAD milking cows’ records. Other confounders could include the type of disease, breed, parity number, energy status and stage of lactation of the cow. However, as can be seen in Table 1, the number of sick cases in our dataset is very low; therefore, future studies with larger databases that include more sick cases should be conducted. The purpose of this study was to examine the feasibility of identifying whether a cow of any type of breed, milk frequency or with different types of farming regimes had a disease by only using MIR spectra data with machine learning methods. Notwithstanding, future studies could consider including the possible confounder effects, such as breed or stage of lactation, to perform the classification of illness using a larger dataset.

## 4. Conclusions

The study explored the potential for milk MIR spectra to be used to identify the health status of a cow by using machine learning methods. Our results indicate that the identification of a cow having some type of disease can be made with a reasonable level of accuracy by considering different breeds of cows, different milking frequencies, lactation status and other factors with a neural network model. However, future studies should be carried out using a larger number of cows and herds and including possible covariates in the model, which would allow the methods to learn better the patterns present in the milk MIR spectrum.

## Figures and Tables

**Table 1:** Number and proportions of grazing cows that were diagnosed as negative and positive for any health problem, lameness, mastitis, reproductive and calving disorders and other ailments during the lactation (early, mid and lactation) at two dairy farms during the 2016 production season.

	Negative Cases ^1^ (N)	Positive Cases ^2^ (N)	Negative Cases (%)	Positive Cases (%)
Any health problem	1806	103	94.60	5.40
Lameness	1897	12	99.37	0.63
Mastitis	1883	26	98.64	1.36
Reproductive disorder ^3^	1849	60	96.86	3.14
Calving disorder	1908	1	99.95	0.05
Other ailments	1907	2	99.90	0.1

^1^ These values correspond to the cows that were healthy; in lameness, for the cows that did not have lameness, some of them were healthy and others had illness such as mastitis, reproductive disorder, etc.; in mastitis, for the cows that did not have mastitis, some of them were healthy and others had illnesses such as lameness, reproductive disorder, etc. ^2^ These values correspond to the cows that had any health problem; the cows that had mastitis; the cows that had lameness. ^3^ Main reproductive disorders include vaginal discharge, metritis, anoestrous and abnormal cycling.

**Table 2:** Performance of classification models obtained in 10 Monte Carlo cross-validation for classifying any health problem and healthy cows during lactation (early, mid and lactation) at two dairy farms during the 2016 production season ^1^.

Models ^2^	Sensitivity	Specificity	Accuracy	PPV	NPV	AUC	MCC
PLS-DA	65.60 ± 5.97	79.59 ± 2.36	78.85 ± 2.23	15.25 ± 3.07	97.66 ± 0.5	72.59 ± 3.27	0.24 ± 0.04
RF	46.22 ± 8.62	79.26 ± 2.15	77.51 ± 1.75	10.94 ± 1.88	96.38 ± 0.73	62.74 ± 3.78	0.14 ± 0.04
SVM	66.39 ± 6.80	76.39 ± 2.92	75.84 ± 2.42	13.48 ± 1.62	97.61 ± 0.61	71.39 ± 2.37	0.22 ± 0.02
NN	61.74 ± 15.99	97.00 ± 2.85	95.16 ± 3.26	59.99 ± 26.20	97.87 ± 0.87	79.37 ± 9.16	0.58 ± 0.22
CNN	57.02 ± 12.70	92.5 ± 5.27	90.63 ± 4.98	33.82 ± 13.41	97.5 ± 0.75	74.76 ± 6.88	0.39 ± 0.13
ESA	57.15 ± 12.38	87.61 ± 6.19	86.02 ± 6.21	24.06 ± 13.07	97.36 ± 0.77	72.38 ± 8.48	0.31 ± 0.16
ESMJ	60.75 ± 5.98	83.57 ± 2.56	82.36 ± 2.27	17.18 ± 3.21	97.46 ± 0.55	72.16 ± 2.9	0.25 ± 0.04
ESWA	56.43 ± 14.56	85.13 ± 7.41	83.61 ± 7.36	21.33 ± 14.18	97.22 ± 0.97	70.78 ± 9.71	0.27 ± 0.17

^1^ These values correspond to the mean ± SD obtained by 10-fold Monte Carlo cross-validation for classifying any health problem (lameness, mastitis, reproductive disorder, etc.). From the cows’ records, the positive cases were cows that had any illness (lameness, mastitis, reproductive disorder, etc.) and negative cases were cows who were healthy (no diagnosed disease); SD = Standard deviation; PPV = positive predicted value; NPV = negative predicted value; AUC = area under the receiver operating characteristic curve; MCC = Matthews correlation coefficient. ^2^ Models used to perform the classification: PLS-DA = partial least squares discriminant analysis, RF = random forest, SVM = support vector machine, NN = neural network, CNN = convolutional neural network, ESA = ensemble stacking average, ESMJ = ensemble stacking major voting and ESWA = ensemble stacking weighted average.

**Table 3:** Performance of normalised Matthews correlation coefficient (nMCC) obtained in 10 Monte Carlo cross-validation for classifying any health problem, lameness and mastitis in grazing cows during lactation (early, mid and lactation) at two dairy farms during the 2016 production season.

Models ^2^	nMCC ^1^
PLS-DA	0.62
RF	0.57
SVM	0.61
NN	0.79
CNN	0.69
ESA	0.65
ESMJ	0.63
ESWA	0.64

^1^ Values corresponding to the normalised Matthews correlation coefficient obtained by the models in classifying any health problem (lameness, mastitis, reproductive disorder, etc.). ^2^ Models used to perform the classification: PLS-DA = partial least squares discriminant analysis, RF = random forest, SVM = support vector machine, NN = neural network, CNN = convolutional neural network, ESA = ensemble stacking average, ESMJ = ensemble stacking major voting and ESWA = ensemble stacking weighted average.

## Data Availability

All relevant data are within the paper. The link for the codes used in the analysis is https://github.com/brendacontla/Identifying-health-status-in-grazing-dairy-cows-from-milk-mid-infrared-spectroscopy-by-using-machine.git (accessed on 30 October 2020).

## Supplementary Materials

The following are available online at https://www.mdpi.com/article/10.3390/ani11082154/s1, Table S1: Performance of the classification models obtained in 10 Monte Carlo cross-validation for predicting any health problem and healthy cows milked once a day at Massey University Dairy 1 farm during the 2016 production season, Table S2: Performance of the classification models obtained in 10 Monte Carlo cross-validation for predicting any health problem and healthy cows milked twice a day at Massey University Dairy 4 farm during the 2016 production season
