# Supplementary material for: Identifying Health Status in Grazing Dairy Cows from Milk Mid-Infrared Spectroscopy by Using Machine Learning Methods
Source: Animals (Basel). 2021 Jul 21;11(8):2154. doi: 10.3390/ani11082154 (PMC8388516; doi:10.3390/ani11082154)
Supplement: Supplementary file 1 [file animals-11-02154-s001.zip › animals-1281582-supplementary.pdf]

Supplementary Materials

Table S1. Performance of the classification models obtained in 10 Monte Carlo cross-validation for classifying any health problem and healthy cows during lactation (early, mid and lactation) at Dairy 1 (OAD) during the 2016 production season [1]

Models[2]	Sensitivity	Specificity	Accuracy	PPV	NPV	AUC	MCC
PLS-DA	51.35 ± 18.7	84.5 ± 3.05	82.52 ± 3.21	15.81 ± 4.35	96.54 ± 1.86	67.93 ± 9.32	0.21 ± 0.09
RF	80.8 ± 9.82	41.02 ± 7.75	43.2 ± 7.62	7.59 ± 2.48	97.47 ± 1.27	60.91 ± 5.79	0.1 ± 0.06
SVM	44.73 ± 17.24	86.36 ± 5.7	83.88 ± 4.16	15.82 ± 6.43	96.31 ± 1.86	65.55 ± 6.84	0.19 ± 0.09
NN	65.34 ± 16.99	98.83 ± 1.53	96.94 ± 2.71	80.33 ± 19.34	97.92 ± 1.49	82.09 ± 9.02	0.71 ± 0.19
CNN	84.46 ± 13.05	91.62 ± 4.75	91.22 ± 4.86	39.68 ± 14.68	98.96 ± 0.96	88.04 ± 7.83	0.54 ± 0.15
ESA	85.85 ± 18.19	65.55 ± 9.29	66.6 ± 9.56	13.13 ± 5.13	98.38 ± 2.92	76.64 ± 10.27	0.24 ± 0.13
ESMJ	75.94 ± 12.82	83.29 ± 2.37	82.93 ± 2.21	20.91 ± 6.3	98.39 ± 0.94	79.61 ± 6.28	0.34 ± 0.08
ESWA	85.44 ± 16.42	68.29 ± 10.59	69.18 ± 10.6	14.32 ± 5.68	98.47 ± 2.36	76.86 ± 12.05	0.26 ± 0.13

[1] These values correspond to the mean ± SD obtained by 10-fold Monte Carlo cross-validation for classifying any health problem (lameness, mastitis, reproductive disorder, etc.). From the cows' records, the positive cases were cows that had any illness (lameness, mastitis, reproductive disorder, etc.) and negative cases were cows who were healthy at Dairy 1 (no diagnosed disease); SD = Standard deviation; PPV = positive predicted value; NPV = negative predicted value; AUC = area under the receiver operating characteristic curve; MCC = Matthews correlation coefficient. [2] Models used to perform the classification: PLS-DA = partial least squares discriminant analysis, RF = random forest, SVM = support vector machine, NN = neural network, CNN = convolutional neural network, ESA = ensemble stacking average, ESMJ = ensemble stacking major voting and ESWA = ensemble stacking weighted average.

Table S2. Performance of the classification models obtained in 10 Monte Carlo cross-validation for classifying any health problem and healthy cows during lactation (early, mid and lactation) at Dairy 4 (OAD) during the 2016 production season [1]

Models[2]	Sensitivity	Specificity	Accuracy	PPV	NPV	AUC	MCC
PLS-DA	80.59 ± 9.61	74.94 ± 2.62	75.23 ± 2.5	16.3 ± 3.32	98.4 ± 0.88	77.77 ± 4.97	0.28 ± 0.05
RF	67.58 ± 13.08	74.3 ± 2.92	73.79 ± 2.32	16.11 ± 4.44	96.88 ± 1.59	70.94 ± 5.87	0.23 ± 0.07
SVM	80.77 ± 8.77	71.62 ± 3.02	72.17 ± 2.52	17.3 ± 4.35	98.03 ± 1.18	76.19 ± 3.43	0.28 ± 0.04
NN	67.93 ± 15.19	93.93 ± 4.62	92.04 ± 4.79	49.51 ± 18.87	97.42 ± 1.49	80.93 ± 8.75	0.53 ± 0.18
CNN	51.27 ± 15.4	94.85 ± 5.62	91.79 ± 5.69	49.27 ± 16.54	96.27 ± 1.57	73.06 ± 9.3	0.45 ± 0.17
ESA	63.99 ± 17.95	86.57 ± 3.82	84.98 ± 3.93	26.32 ± 8.94	96.97 ± 1.67	75.28 ± 9.35	0.34 ± 0.12
ESMJ	74.77 ± 9.31	75.95 ± 3.27	75.79 ± 2.75	18.59 ± 4.47	97.56 ± 1.3	75.36 ± 3.96	0.28 ± 0.05
ESWA	64.46 ± 19.19	82.84 ± 4.23	81.53 ± 4.61	21.75 ± 7.34	96.81 ± 1.92	73.65 ± 10.45	0.29 ± 0.13

[1] These values correspond to the mean ± SD obtained by 10-fold Monte Carlo cross-validation for classifying any health problem (lameness, mastitis, reproductive disorder, etc.). From the cows' records, the positive cases were cows that had any illness (lameness, mastitis, reproductive disorder, etc.) and negative cases were cows who were healthy at Dairy 4 (no diagnosed disease); SD = Standard deviation; PPV = positive predicted value; NPV = negative predicted value; AUC = area under the receiver operating characteristic curve; MCC = Matthews correlation coefficient. [2] Models used to perform the classification: PLS-DA = partial least squares discriminant analysis, RF = random forest, SVM = support vector machine, NN = neural network, CNN = convolutional neural network,

ESA = ensemble stacking average, ESMJ = ensemble stacking major voting and ESWA = ensemble stacking weighted average.
